# Impact of gender on clinical characteristics in patients with atrial fibrillation and STEMI

**DOI:** 10.25122/jml-2025-0041

**Published:** 2025-07

**Authors:** Dan Pasaroiu, Imre Benedek, Andra Chirtes, Violeta Masca, Renata Gerculy, Monica Chitu, Theodora Benedek

**Affiliations:** 1 Clinic of Cardiology, County Emergency Clinical Hospital, Targu Mureș, Romania; 2 Center of Advanced Research in Multimodality Cardiac Imaging, CardioMed Medical Center, Targu Mures, Romania; 3 Doctoral School of Medicine and Pharmacy, University of Medicine, Pharmacy, Science and Technology George Emil Palade of Targu Mures, Targu Mures, Romania

**Keywords:** gender, atrial fibrillation, STEMI, heart failure, NT-proBNP

## Abstract

Atherosclerotic cardiovascular disease remains one of the leading causes of morbidity and mortality worldwide, accounting for approximately 3.9 million deaths annually due to its complications. This single-center, retrospective cohort study included 109 patients who underwent coronary percutaneous coronary intervention (PCI) for ST-elevation myocardial infarction (STEMI) between April and July 2022 at the Cardiology Clinic of the County Emergency Clinical Hospital in Targu Mureș, Romania. Women diagnosed with STEMI were found to be older at the time of presentation compared to men, with this difference reaching statistical significance (*P* = 0.0148). The incidence of atrial fibrillation was higher in women, with a statistically significant difference (*P* = 0.0258). Stratification of patients based on the location of culprit lesions did not reveal a statistically significant difference in the occurrence of atrial fibrillation. N-terminal pro-brain natriuretic peptide (NT-proBNP) levels were significantly higher in women than in men (*P* = 0.0200). The multivariate analysis revealed that the occurrence of atrial fibrillation was dependent on age (*P* = 0.0025), smoking (*P* < 0.0001), and cholesterol levels (*P* = 0.0182), but independent of sex (*P* = 0.2094), ejection fraction (*P* = 0.2293), the presence of hypertension (*P* = 0.1142), chronic kidney disease (*P* = 0.6935), diabetes mellitus (*P* = 0.9375), triglyceride levels (*P* = 0.7614), high-sensitivity cardiac troponin I (hs-cTnI, *P* = 0.4422), and creatine kinase (CK, *P* = 0.7420). In summary, women with STEMI presented at an older age, had higher NT-proBNP levels, experienced more frequent atrial fibrillation, and had a greater likelihood of circumflex artery involvement. Smoking and age were the only factors significantly associated with atrial fibrillation in the multivariable analysis.

## INTRODUCTION

Atherosclerotic disease is one of the most common and dangerous illnesses globally, significantly contributing to morbidity and mortality, with an estimated 3.9 million deaths annually attributed to its complications [1]. The condition is characterized by the accumulation of cholesterol and inflammatory cells within arterial walls, leading to vessel narrowing, increased arterial stiffness, and reduced blood flow [2]. Despite a reduction in mortality rates across European nations, including Central and Eastern Europe, the prevalence of atherosclerotic disease is increasing. Atherosclerotic disease constitutes a major etiological factor in myocardial infarction, stroke, and heart failure, resulting in marked reductions in quality of life and life expectancy while exerting substantial pressure on healthcare infrastructures. Acute myocardial infarction with elevated ST segment (STEMI) stands for the most critical and life-threatening condition due to its associated complications [3].

Atrial fibrillation (AF) is increasingly recognized in patients with acute coronary syndrome (ACS), causing the distinction between two categories: patients with pre-existing AF and those who develop new-onset AF (NOAF) concomitant with the acute ischemic event [4]. The reported incidence of NOAF during ACS ranges from 2.3% to 37% in the literature, with rates during STEMI specifically varying between 5% and 14% [5,6]. While substantial data and studies exist regarding the management of ACS in patients with known AF [7,8], there remains a limited understanding of the optimal management of patients with NOAF, particularly during the acute phase of ACS and STEMI.

The etiology of NOAF appears to be multifactorial, with numerous factors acting as triggers or contributing to the arrhythmic episode, including atrial ischemia and dilation, autonomic nervous system dysregulation (characterized by increased sympathetic activity and reduced vagal tone), systemic and localized inflammation, and hormonal activation [9]. These mechanisms are based on structural and electrical remodeling of the atria, promoting the development of NOAF. Identified risk factors for NOAF include common cardiovascular risk factors such as age, sex, obesity, and smoking, as well as conditions like hypertension, diabetes, chronic kidney disease, and prior arrhythmias. Clinical parameters such as reduced ejection fraction (EF) have also been associated with a higher risk of NOAF [10,11].

Left atrial coronary branch involvement in ACS is correlated with the onset of AF. The coronary arteries supplying the left atrium have been described as originating from the proximal segment of the circumflex artery [12,13], and even circumflex artery obstruction detected with coronary computed tomography (CCT) was associated with an increased risk of AF recurrence after persistent AF [14].

Timely reperfusion is essential in the management of STEMI to minimize myocardial injury and improve clinical outcomes. The diagnosis-to-needle time (DNT) serves as a critical performance indicator, reflecting the efficiency of diagnostic and therapeutic interventions in emergency cardiac care. Delays in DNT may arise from pre-hospital factors, diagnostic inaccuracies, or logistical inefficiencies within the emergency department. Current research emphasizes the need to optimize protocols, enhance interdepartmental coordination, and leverage advanced diagnostic technologies to reduce DNT. Such improvements are pivotal in achieving superior clinical outcomes and decreasing mortality rates among STEMI patients [15].

In the Vienna STEMI network, unadjusted short- and long-term mortality rates were higher in women compared to men treated for STEMI. This disparity is primarily attributed to differences in patient characteristics, including older age and a higher burden of comorbidities in women, as well as longer patient-related delays in seeking treatment [16].

The aim of the present study was to investigate gender-based differences in the occurrence of atrial fibrillation in STEMI patients, focusing on gender distribution and the specific coronary territory that underwent revascularization.

## MATERIAL AND METHODS

A single-center, retrospective cohort study was conducted involving 109 patients who underwent percutaneous coronary intervention (PCI) for STEMI between April 2022 and July 2022 at the Cardiology Clinic of Targu Mureș County Emergency Clinical Hospital.

### Study population

Clinical and demographic data were retrospectively compiled into a database. Patients were stratified by sex: Group 1 consisted of 28 women, and Group 2 consisted of 81 men. The study was conducted in accordance with institutional guidelines, national legal requirements, European standards, and the revised Declaration of Helsinki. The inclusion criteria were patients presenting with STEMI who underwent PCI. Exclusion criteria included myocardial infarction with non-obstructive coronary arteries (MINOCA), myocarditis, advanced coronary artery disease (CAD) requiring surgical revascularization (coronary artery bypass grafting), and patient refusal to participate in the study.

STEMI was diagnosed using a 12-lead electrocardiogram (ECG) showing ST-segment elevation in two or more contiguous leads or a new left bundle branch block (LBBB). Diagnostic criteria included J-point elevation greater than 2 mm (0.2 mV) in leads V2 and V3, and at least 1 mm in other leads (2.5 mm for men under 40 years; 1.5 mm for women), or the presence of new or presumed LBBB, accompanied by elevated cardiac troponin levels exceeding the 99^th^ percentile upper reference limit. The diagnosis of diabetes was determined based on a history of the condition, with or without the use of medication, fasting blood glucose levels exceeding 126 mg/dL, or glycosylated hemoglobin levels greater than 6.5%. Hypercholesterolemia was defined as a documented history of the condition, current chronic treatment with any cholesterol-lowering medication at the time of admission, or fasting total cholesterol levels exceeding 200 mg/dL. Smoking status was classified as either current or prior, based on the patient's history prior to admission to the hospital. Atrial fibrillation was identified using either an ECG or 24-hour Holter monitoring. Hemodynamic instability was documented, and urgent pharmacological intervention was provided when necessary. N-terminal pro-brain natriuretic peptide (NT-proBNP) levels were measured, with values exceeding 300 pg/mL deemed clinically significant in patients with sinus rhythm and values above 600 pg/mL considered significant in those with atrial fibrillation. Ejection fraction was evaluated via standard transthoracic echocardiography. Ventricular arrhythmias were defined as ventricular fibrillation or sustained ventricular tachycardia requiring electrical cardioversion or antiarrhythmic treatment. Total creatine kinase (CK) concentration was quantified.

### Urgent coronary angiography

Angiographic imaging was conducted using the Philips Azurion 7 B20 system (Veenpluis, Netherlands).

Patients diagnosed with STEMI were transferred to our catheterization laboratory, where a 6-French sheath was inserted into either the radial, brachial, or femoral artery. Prior to the procedure, heparin was administered at a dose of 60-80 IU/kg. Revascularization of all lesions responsible for the STEMI was performed in line with established clinical guidelines. Following the procedure, the arterial sheath was removed. Hemostasis was achieved using a hemostasis device for the radial artery, or manual compression for the brachial and femoral arteries, followed by the application of a compression bandage. The mean length of hospitalization ranged from 6 to 10 days. To mitigate the risk of contrast-induced nephropathy, adequate hydration was maintained. In addition to standard treatment for acute coronary syndrome, patients received individualized therapy based on their lipid profile, including total cholesterol, low-density lipoprotein (LDL), high-density lipoprotein (HDL), and triglyceride levels.

### Statistical analysis

Statistical analysis was conducted using GraphPad Prism 9.3.0 software (GraphPad Software Inc., San Diego, USA). Prior to analysis, all data were evaluated for normality. The results are presented as counts and percentages. A *P* value of less than 0.05 was considered statistically significant.

## RESULTS

### Study population

The multivariate analysis revealed that the occurrence of atrial fibrillation was dependent on age (*P* = 0.0025), smoking (*P* < 0.0001), and cholesterol levels (*P* = 0.0182), but independent of sex (*P* = 0.2094), ejection fraction (*P* = 0.2293), the presence of hypertension (*P* = 0.1142), chronic kidney disease (*P* = 0.6935), diabetes mellitus (*P* = 0.9375), triglyceride levels (*P* = 0.7614), hs-cTnI (*P* = 0.4422), and CK (*P* = 0.7420).

**Figure 1:**
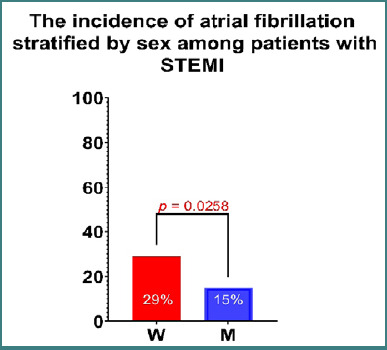
The incidence of atrial fibrillation stratified by sex among patients with STEMI. A statistically significant higher prevalence was observed in the female group (P = 0.0258). W, women; M, men.

**Figure 2:**
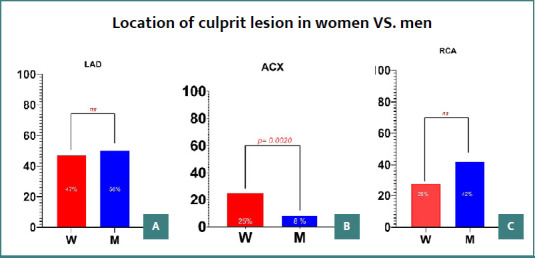
Location of culprit lesion in women vs. men. A, Prevalence of culprit lesions in the left anterior descending artery (LAD) stratified by gender; B, Prevalence of culprit lesions in the circumflex artery (ACX), stratified by gender, with a difference reaching statistical significance; C, Prevalence of culprit lesions in the right coronary artery stratified (RCA), stratified by gender; W, women; M, men;

Women diagnosed with STEMI were found to be older at the time of presentation compared to men, with this difference reaching statistical significance (*P* = 0.0148) (Table 1). A higher incidence of atrial fibrillation was observed in women, with the difference reaching statistical significance (*P* = 0.0258; relative risk [RR]: 1.933; 95% CI, 1.121–3.379) (Figure 1). Furthermore, the culprit lesion was more frequently located in the circumflex artery in women (*P* = 0.0020; RR: 3.125; 95% CI, 1.523–6.540) (Figure 2). There were no significant differences in the prevalence of culprit lesions between the left anterior descending artery and the right coronary artery.

**Figure 3:**
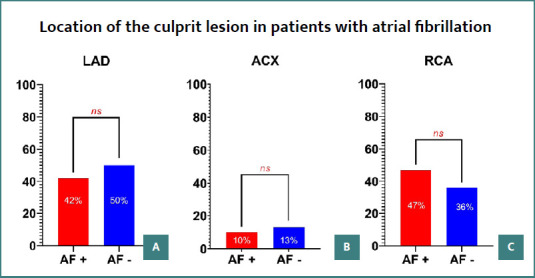
Location of culprit lesion in patients with atrial fibrillation. A, Atrial fibrillation in patients with STEMI and culprit lesions in the left anterior descending artery; B, Atrial fibrillation in patients with STEMI and culprit lesions in the circumflex artery; C, Atrial fibrillation in patients with STEMI and culprit lesions in the right coronary artery; W, women; M, men; AF+, Patient with atrial fibrillation. AF-, Patient without atrial fibrillation.

Furthermore, stratification of patients based on the location of culprit lesions did not reveal a statistically significant difference in the occurrence of atrial fibrillation (Figure 3).

**Table 1:** Baseline characteristics of the study population

	Women (*n* = 28)	Men (*n* = 81)	*P* value
Age, mean ±SD	66.46 ± 13.01	59.59 ± 12.52	0.0148
Risk factors			
CKD *n* (%)	3 (10.71%)	2 (2.46%)	>0.9999
Hypertension *n* (%)	14 (50%)	43 (54%)	0.6712
Smoking* *n* (%)	6 (21%)	38 (47%)	0.0002
Diabetes *n* (%)	8 (29%)	17 (22%)	0.3304
Ventricular disfunction			
NT-proBNP, mean ±SD pg/ml	4892.91 ± 1970	2348.12 ± 521	0.0200
Ejection fraction %, mean ±SD	39.37 ± 7.88	42 ± 7.96	0.2403
Myocardial necrosis			
Hs-cTnI, mean ±SD pg/ml	10675.44 ± 15123.74	4478.6 ± 8408.19	0.4965
CK, mean ±SD U/L	446.84 ± 458.53	712.45 ± 752.38	0.2349
Dyslipidemia *n* (%)	11 (39.28%)	55 (67.9%)	0.3083
Cholesterol, mean ±SD mg/dl	177.4 ± 52.54	168.57 ± 114.46	0.0994
Triglycerides, mean ±SD mg/dl	138.08 ± 85.46	168.57 ± 114.46	0.2760
Culprit lesion localization			
LAD *n* (%)	13 (47%)	41 (50%)	0.7773
LCX *n* (%)	7 (25%)	6 (8%)	0.0020
RCA *n* (%)	8 (28%)	34 (42%)	0.0536

**Figure 4:**
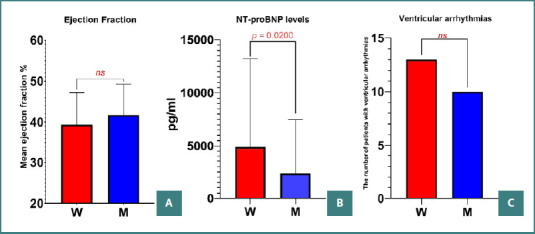
Left ventricular function and cardiac arrhythmia in women vs. men. A, Mean ejection fraction value stratified by gender; B, NT-proBNP levels highlighting a statistically significant difference in female patients; C, Stratification of ventricular arrhythmia occurrence by gender. W, women; M, men;

SD, Standard Deviation; Ns, Not Significant; CKD, Chronic Kidney Disease; Hs-Ctni, High-Sensitivity Troponin I; CK, Total Creatine Kinase; LAD, Left Anterior Descending Coronary Artery; LCX, Circumflex Coronary Artery; RCA, Right Coronary Artery; * Present Or Past;

There were no statistically significant differences in the occurrence of ventricular arrhythmias between the groups (*P* = 0.6584). No statistically significant difference was found in the ejection fraction between the two groups (*P* = 0.2403); both groups had approximately the same mean ejection fraction value. Additionally, NT-proBNP levels were significantly higher in women compared to men (*P* = 0.0200) (Figure 4).

## DISCUSSION

This study aimed to evaluate gender-based differences among STEMI patients, with a particular focus on the association between atrial fibrillation and heart failure, as determined by NT-proBNP levels, and the impact of revascularization procedures in the circumflex artery. Our findings highlight several significant gender-related disparities, underscoring the need for gender-specific strategies in the management of STEMI.

One key finding was that women presented with STEMI at an older age than men. This aligns with previous studies that have shown a later presentation in women. The difference may result from delayed symptom recognition and unique pathophysiological factors in women [15,17]. Since age is a risk factor for worse STEMI outcomes, this could partly explain the poorer prognosis observed in women [18].

Our study revealed significantly higher NT-proBNP levels in women compared to men. This finding aligns with other studies showing elevated levels of this biomarker in women with heart failure and acute coronary syndromes [19,20]. Elevated NT-proBNP is linked to worse cardiac function and predicts adverse outcomes. However, no significant difference in left ventricular dysfunction was observed.

Women more often develop heart failure, including heart failure with preserved ejection fraction. Enhanced concentric ventricular remodeling in women may explain the greater declines in systolic and diastolic function that occur with aging. Similar gender differences in arterial remodeling have also been reported [21].

Although high-sensitivity troponin levels were higher at admission in women, suggesting a later presentation, the difference between the groups did not reach statistical significance. Furthermore, creatine kinase levels were higher in the male group, indicating more extensive myocardial necrosis, although the difference did not reach statistical significance. In contrast, the female group exhibited more pronounced myocardial dysfunction, suggesting mechanisms other than necrosis, considering that no statistically significant difference was found when analyzing the distribution of ejection fractions. Additionally, no statistically significant difference was observed in cholesterol levels or the incidence of diabetes mellitus between the two groups. Although the multivariate analysis highlighted a correlation between smoking and the occurrence of AF, in the studied population, AF was more prevalent among women, despite fewer of them being smokers.

Additionally, we observed a greater proportion of AF in women, which is consistent with previous studies showing that women are at a higher risk of developing NOAF during ACS or STEMI [22]. The incidence of AF in women who had the culprit lesion located in the circumflex artery during ACS has been a subject of increasing interest. Studies indicate that women with STEMI and circumflex artery involvement are at a higher risk for developing AF compared to men. The circumflex artery supplies blood to the left atrium, and ischemia in this region can promote atrial remodeling, predisposing women to NOAF. Studies have shown that women are more likely to experience AF during STEMI. This is particularly common when the culprit lesion is in the circumflex artery. Gender-related differences in myocardial response to ischemia, hormonal influences, and autonomic nervous system activity may contribute to this phenomenon [23]. While the prevalence of atrial fibrillation was higher in women, no statistically significant difference was found when the prevalence of atrial fibrillation was analyzed according to the location of the culprit lesion. Revascularization of the circumflex artery may help reduce the ischemic burden and lower the incidence of AF; however, the risk remains elevated in women due to factors such as delayed presentation and treatment initiation [24]. Interestingly, no significant differences were observed in the levels of high-sensitivity troponin, creatine kinase, or the incidence of ventricular arrhythmias between the sexes. This suggests that while women may present with a more advanced disease state and increased biomarker levels, the acute myocardial injury in both genders appears to be similar in terms of its biochemical markers and ventricular arrhythmic complications. This finding is consistent with studies that show similar mortality rates related to STEMI in both men and women after adjusting for age and comorbidities [25].

While previous studies have identified additional risk factors for atrial fibrillation in patients with ischemia, such as a higher prevalence among women, an increased burden of cardiovascular risk factors, severe left ventricular dysfunction, and the presence of ≥2 diseased atrial branches, the current study found through multivariate analysis that only smoking status and age were significant predictors of atrial fibrillation. It is plausible that a larger patient cohort might be required in this study to achieve statistical significance for the other risk factors [26,27].

### Limitation

While our study provides important insights, several limitations must be acknowledged. The retrospective design, relatively small sample size, limited inclusion of women in the database, and the focus on a single center may limit the generalizability of our findings. Future prospective studies, ideally multicenter trials, with larger sample sizes, are needed to confirm these findings and further explore the gender-specific mechanisms driving these differences.

## CONCLUSION

In conclusion, our study highlights gender-based differences in STEMI patients, with women presenting at an older age, higher NT-proBNP levels, more frequent atrial fibrillation, and a greater likelihood of circumflex artery involvement. Despite similar left ventricular function and ejection fraction, alternative mechanisms may contribute to heart failure in women. Smoking and age were the only factors significantly associated with atrial fibrillation in the multivariable analysis, but larger cohorts are needed to validate broader risk factors.
